# Lipase-Catalyzed Strategies for the Preparation of Enantiomeric THIQ and TH*β*C Derivatives: Green Aspects

**DOI:** 10.3390/molecules28176362

**Published:** 2023-08-30

**Authors:** György Orsy, Enikő Forró

**Affiliations:** Institute of Pharmaceutical Chemistry, University of Szeged, Eötvös u. 6, H-6720 Szeged, Hungary; orsy.gyorgy@szte.hu

**Keywords:** continuous-flow reactions, enzymatic *O*-acylation, enzymatic *N*-acylation, enzymatic CO*OEt* hydrolysis, green solvents, primary hydroxy group, secondary amino group, TH*β*C, THIQ

## Abstract

This report reviews the most important lipase-catalyzed strategies for the preparation of pharmaceutically and chemically important tetrahydroisoquinoline and tetrahydro-*β*-carboline enantiomers through *O*-acylation of the primary hydroxy group, *N*-acylation of the secondary amino group, and CO*OEt* hydrolysis of the corresponding racemic compounds with simple molecular structure, which have been reported during the last decade. A brief *introduction* describes the importance and synthesis of tetrahydroisoquinoline and tetrahydro-β-carboline derivatives, and it formulates the objectives of this compilation. The strategies are presented in chronological order, classified according to function of the reaction type, as kinetic and dynamic kinetic resolutions, in the *main text*. These reactions result in the desired products with excellent *ee* values. The pharmacological importance of the products together with their synthesis is given in the *main text*. The enzymatic hydrolysis of the hydrochloride salts as racemates of the starting amino carboxylic esters furnished the desired enantiomeric amino carboxylic acids quantitatively. The enzymatic reactions, performed in *t*BuOMe or H_2_O as usable solvents, and the transformations carried out in a continuous-flow system, indicate clear advantages, including atom economy, reproducibility, safer solvents, short reaction time, rapid heating and compression vs. shaker reactions. These features are highlighted in the *main text*.

## 1. Introduction

Derivatives containing a tetrahydroisoquinoline (THIQ) or tetrahydro-β-carboline (TH*β*C) core are important from both biological and chemical aspects. A large number of naturally occurring THIQs have significant pharmacological activity [1,2]. For example, (*R*)-salsolidine (*Genista pungens*) has MAO A inhibitory effect [3], whereas expectorant emetine, (*Ipecacuanhe*) [4], antitussive noscapine (*Papaver somniferum*) [5], and Trabectidine, approved as Yondelis^®^ (*Ecteinascidia turbinate*) [6], show anticancer effect. Saframycine (*Myxococcus xanthus*) [7] and liensinine (*Nelumbo nucifera*) [8] exhibit pharmaceutical activities toward HIV and cancer. Some synthetic THIQ alkaloid analogues, such as Zalypsis^®^ [9], possess anticancer activity. The use of TH*β*C-containing compounds in medicine is as important as in the case of the abovementioned THIQ derivatives. For instance, vincristine, vinblastine [10], and reserpine [11] show antihypertensive and/or antitumor activity. Harmicine (*Kopsia griffithii*) [12] has antinociceptive activity, whereas (+)-7-bromotypargine (*Ancorina* sp.) [13] shows antimalarial activity. Synthetic TH*β*Cs are used in drug research. For instance, Tadalafil (Cialis^®^) [14] was successfully applied in the treatment of erectile dysfunction. MK-4256 [15] is an antagonist of somatostatin subtype receptor 3 and it was a promising preclinical candidate for the medication of type II diabetes mellitus. Significant proliferation inhibitor effect of pyridoxal TH*β*C derivatives against *Plasmodium falciparum* was also reported [16]. In addition, synthetic 1-substituted *N*-acylated TH*β*Cs exhibits inhibitory effect against the Breast Cancer Resistance Protein (ABCG2) [17]. The protected form of (*S*)-1-isopropyl-TH*β*C was used in the synthesis of (*S*)-quinolactacin-B with activity against tumor necrosis factor [18].

Therefore, it is not surprising that a large number of publications describing the preparation of important THIQ and TH*β*C derivatives have appeared and most of them reviewed [1,2,19,20,21,22,23]. Indeed, only very few articles describe the lipase-catalyzed synthesis of THIQ and TH*β*C enantiomers, with simple molecular structures.

Lipases (triacylglycerol hydrolases E.C. 3.1. 1.3) are versatile enzymes, widely used for various kinds of biocatalyzed reactions, performed in both aqueous and nonaqueous media. They can catalyze chemical transformations with high catalytic efficiency and specificity as chemo-, regio-, and enantio-selectivities. Lipase-catalyzed enantioselective acylation of alcohols and amines together with the hydrolysis of esters and amides are among the most utilized biocatalytic methods for the preparation of enantiomerically pure alcohols, esters, amines, amides, and acids. The reason is that lipases are highly active and robust enzymes with broad substrate specificities.

From a green chemistry perspective, the best solvent for reactions is no solvent [24,25,26,27,28]. The solvent-free reactions are not always achievable and the selection of another more efficient solvent becomes critical. Water has increasing popularity, because it is inexpensive, nontoxic, nonflammable, and safe for use. In addition, to ensure high activity and selectivity, water allows easy product isolation by filtration.

Green solvents play a crucial role in lipase-catalyzed reactions, offering numerous advantages over traditional solvents in terms of environmental sustainability, safety, and efficiency [29,30,31,32,33,34,35]. Lipase-catalyzed reactions are widely employed in various industries, including the production of pharmaceuticals, food, and biofuels, due to their ability to produce enantiopure compounds and minimize unwanted side reactions. The choice of solvent significantly influences the overall performance of the enzymatic reaction, and the use of green solvents has emerged as a preferred option for several reasons. First, green solvents are available from renewable resources, making them inherently more sustainable compared to conventional solvents obtained from petroleum-based sources. This is in accordance with the growing global emphasis on reducing the carbon footprint and moving toward ecofriendly practices in chemical processes. Furthermore, green solvents are nontoxic and biodegradable, minimizing environmental hazards and reducing health risks for both workers and consumers. Their low toxicity is also beneficial for the stability and activity of enzymes during catalysis leading to enhanced reaction rates and product yields. Moreover, the use of green solvents promotes improved enzymatic selectivity. They typically have lower polarity, which can reduce nonspecific interactions between the enzyme and the substrate, resulting in higher enantioselectivity and regioselectivity. This is particularly important in the synthesis of pharmaceutical intermediates and fine chemicals, where chirality is of utmost significance. Green solvents also contribute to overcome mass transfer limitations. They possess lower viscosities compared to conventional solvents, facilitating better diffusion of substrates to the active site of the enzyme and faster product release. These properties can lead to shorter reactions and increased productivity. Furthermore, green solvents are often compatible with a wide range of substrates and enzyme types. Their ecofriendly nature, reduced toxicity, improved selectivity, and enhanced mass transfer properties make them invaluable tools for sustainable and efficient enzymatic processes. Although there are some greener organic solvents, for example, cyclopentyl methyl ether (CPME), a promising ecofriendly solvent without any handling difficulties, and *t*BuOMe, which is one of the most widely used solvents for lipase-catalyzed transformations. Note, however, that according to the green solvent selection guide [36], there are some unfavorable issues and substitution is advisable.

The majority of lipase-catalyzed reactions have been performed as batch reactions. However, enzymatic continuous-flow processes, because of their numerous advantages, have gained significant attention in recent years as promising alternatives to traditional batch reactions [37,38,39,40,41,42]. For example, continuous flow systems allow controlled flow of reactants, ensuring a constant supply of substrates to the enzyme, which improves the overall reaction efficiency, leading to higher productivity and increased yield of the desired products. Enzyme-catalyzed reactions in continuous flow can be finely tuned by adjusting residence times, temperature, and enzyme concentration, enabling better selectivity in product formation, minimizing unwanted byproducts, and reducing the need for downstream purification steps. Continuous-flow reactors can handle hazardous materials more safely than traditional batch reactors. The smaller reaction volumes in continuous flow reduce the risk of accidental spills and exposure to toxic or reactive substances, enhancing overall operator safety. The continuous flow of reactants ensures that they spend less time in the reactor, leading to shorter reaction times. This is of particular significance when working with unstable substrates or sensitive enzymes that may degrade over prolonged exposure. Enzymatic continuous-flow processes are readily scalable, making it easier to transition from laboratory-scale to industrial-scale production. Additionally, continuous-flow setups can be easily integrated into automated systems, allowing precise control and reproducibility.

Although lipase-catalyzed kinetic resolution (KR) methods have been developed for the enantioselective acylation in a large number of primary and especially secondary alcohols or the hydrolysis of their corresponding esters, the first chemoenzymatic method for the preparation of optically active alcohols and esters possessing the 1,2,3,4-tetrahydroquinoline (THQ) moiety was published only recently [43]. The enantioselective transesterification (*E* values > 328) of racemic 1,2,3,4-THQ-propan-2-ols was performed with vinyl acetate (VA) in the presence of immobilized *Candida antarctica* lipase B (CALB) or *Burkholderia cepacia* lipase (BCL) or engineered acyltransferase variants from *Mycobacterium smegmatis* (MsAcT). These reactions furnished (*S*)-alcohols and the corresponding (*R*)-acetates with excellent optical purities (*ee* > 99%).

*Candida rugosa* lipase (AY)-catalyzed KR of 1-methyl THIQ through *N*-alkoxycarbonylation with 3-methoxyphenyl allyl carbonate in water-saturated toluene at 30 °C, resulting in the desired (*R*)-1-methyl THIQ (*ee* 98% and yield 47%) and (*S*)-amide (*ee* 99% and yield 46%) has been described [44].

DKR of 6,7-dihydroxy-1-methyl-1,2,3,4-THIQ by [IrCp*I_2_]_2_ and lipase AY in the presence of 3-methoxyphenyl propyl carbonate in toluene at 40 °C, resulting in (*R*)-carbamate (*ee* 96%, conversion 90% after 23 h) was described [45].

**Scheme 1:**
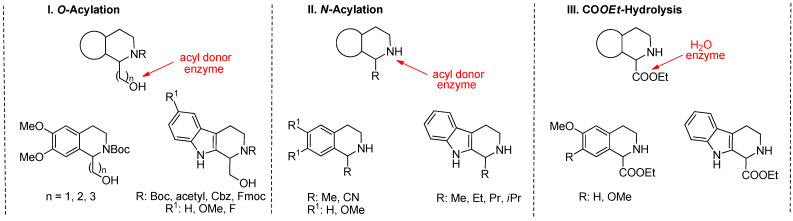
Lipase-catalyzed routes to THIQ and TH*β*C enantiomers.

The aim of this compilation is to present the most important lipase-catalyzed routes devised for the preparation of pharmaceutically and chemically important THIQ and TH*β*C enantiomers (simple molecular structures) with the appropriate absolute configuration of the asymmetric center (Scheme 1) over the last decade. The transformations through *O*-acylation (I), *N*-acylation (II), and CO*OEt* hydrolysis (III) of the corresponding racemates (Scheme 1) are classified as KR (a maximum yield of 50% enantiopure product is obtainable from asymmetric acylation, hydrolysis, etc.) and DKR (this theoretically allows 100% conversion), and they are discussed in chronological order. The reactions, performed under environmentally benign green conditions, are highlighted in the *main text*.

## 2. Enzymatic Strategies for the Synthesis of THIQ and TH*β*C Enantiomers

### 2.1. KR through O-Acylation

Successful lipase-catalyzed methods for the asymmetric *O*-acylation of the primary hydroxy group of several 1,2,3,4-THIQ and 1,2,3,4-TH*β*C racemates were developed. Efficient enzymatic resolution processes of racemates, bearing a stereogenic center separated from the functional group by a larger nonchiral moiety, were also developed. In general, good yields accompanied by high enantioselectivities were obtained, thus underlining the potential of enzymes to recognize and transform enantiomers of racemates with ‘remote’ stereogenic centers in an enantioselective manner. The reactions were performed in an organic solvent either in a continuous-flow system or as a batch reaction.

**Scheme 2:**
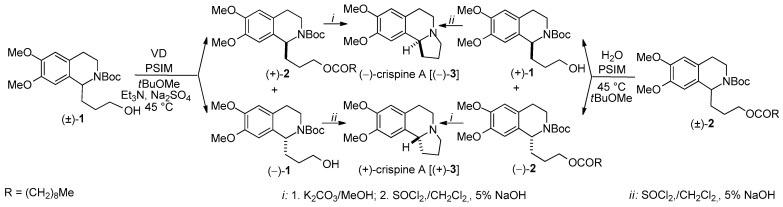
Synthesis of the crispine A enantiomers.

The total synthesis of the antitumor-active alkaloid crispine A [46] enantiomers (*ee* ≥ 94%) has been performed [47] through *Burkholderia cepacia* lipase (PSIM)-catalyzed *S*-selective *O*-acylation of the primary hydroxy group of *N*-Boc-protected 1-(3-hydroxypropyl)-6,7-bis(methyloxy)-1,2,3,4-THIQ ((±)-**1**) (vinyl decanoate (VD), Et_3_N, Na_2_SO_4_, *t*BuOMe as a green solvent, 45 °C) (*E* = 68) or the *R*-selective hydrolysis of the corresponding racemic *O*-decanoate ((±)-**2**, R = (CH_2_)_8_Me, H_2_O, *t*BuOMe, 45 °C) (*E* = 52)) (Scheme 2). The enzymatic resolutions, performed in two steps, afforded key alcohol (+)-**1** and (−)-**1** and ester (+)-**2** and (−)-**2** enantiomers with high *ee* (≥94%, yields ≥ 21%). Ring-closure reactions of these enantiomers with thionyl chloride afforded the desired crispine A enantiomers (+)-**3** and (−)-**3** (*ee* ≥ 95%, yield ≥ 78%). It was mentioned that (+)-**1** and (−)-**1** can also serve as starting enantiomers for the synthesis of enantiomerically pure crispine E [48]. The authors also noted that, ’although crispine A enantiomers have been prepared by asymmetric routes, to the best of our knowledge, they have not yet been obtained via a strategy involving enzyme-catalyzed kinetic resolution’ [47].

**Scheme 3:**
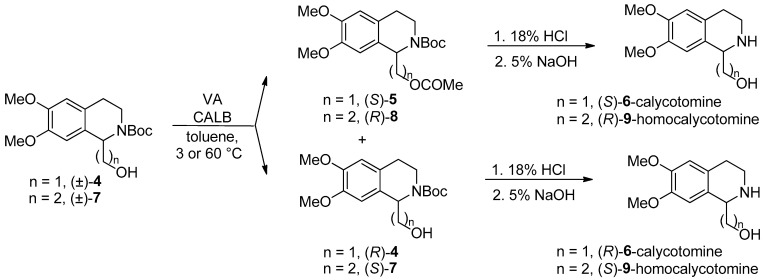
Synthesis of the calycotomine and homocalycotomine enantiomers.

Both enantiomers of calycotomine ((*R*)-**6** and (*S*)-**6**) were prepared [49] through *Candida antarctica* lipase B (CALB)-catalyzed asymmetric *O*-acylation of *N*-Boc-protected (6,7-dimethoxy-1,2,3,4-THIQ-1-yl)methanol ((±)-**4**)). Taking advantage of the continuous-flow technique (atom economy, reproducibility, safer solvents, short reaction time, rapid heating and compression), the authors carried out the preliminary experiments in a continuous-flow system (an H-Cube system in ‘*No H*_2′_ mode, equipped with a stainless-steel enzyme-charged cartridge, under laboratory conditions). The preparative-scale resolution of (±)-**4** was performed as a batch reaction, in an incubator shaker with VA in toluene at 60 °C (Scheme 3) (*E* > 200). The resulting (*R*)-**4** amino alcohol and (*S*)-**5** amino ester, obtained with high enantiomeric excess (*ee* = 99%) were separated by column chromatography (silica, elution with *n-*hexane:EtOAc (5:1)) (yields *≥* 43%), and then transformed into the desired calycotomine (*R*)-**6** and (*S*)-**6** (*ee* = 99%, yields *≥* 73%).

CALB-catalyzed *O*-acylation of *N*-Boc-protected (6,7-dimethoxy-1,2,3,4-THIQ-1-yl)ethanol ((±)-**7**)) has also been described [50]. Preliminary experiments were performed either in a continuous-flow system or as batch reactions, while the preparative-scale resolution was carried out in two steps with VA, Et_3_N, and Na_2_SO_4_ in toluene at 3 °C, as a batch reaction (Scheme 3). The furnished amino alcohol (*S*)-**7** and amino ester (*R*)-**8** (*ee* ≥ 94%) were separated by column chromatography (silica, elution with *n-*hexane:EtOAc (2:1) (yields *≥* 32%) and they were then transformed (treatment with 18% HCl, followed by 5% NaOH) into the desired homocalycotomine (*S*)-**9** (the intermediate of emetine [51]) and (*R*)-**9** (*ee* ≥ 94%, yields *≥* 71%).

A systematic study was carried out in a continuous-flow system on the *O*-acylation of THIQ amino alcohol homologues (±)-**1**, (±)-**4**, and (±)-**7** containing a remote stereogenic center (VA, CALB, toluene, 80 bar, 60 °C, 0.1 mL min^−1^ flow) [50]. The authors found that the enantioselectivity decreased from 200 to 1, when the distance between the hydroxy group and the stereogenic center increased from one carbon atom ((±)-**4**) to three carbon atoms ((±)-**1**).

**Scheme 4:**
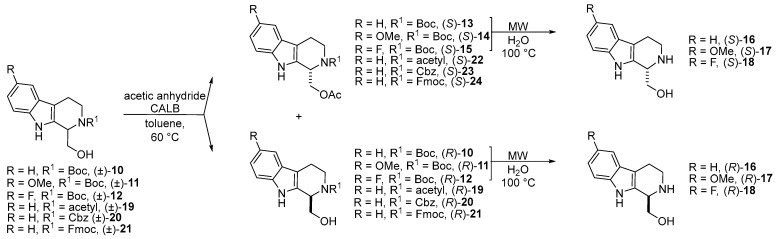
Synthesis of 1-hydroxyalkyl-TH*β*C enantiomers substituted in different manners.

Enantiomeric *N*-Boc-protected amino alcohols ((*R*)-**10**–(*R*)-**12**) and amino esters ((*S*)-**13**–(*S*)-**15**) were prepared through CALB-catalyzed *O*-acylation of the hydroxy group of TH*β*C (±)-**10**, (±)-**11**, and (±)-**12** [52]. The optimization reactions were carried out in a continuous-flow system, while preparative-scale resolutions were performed as batch reactions (incubator shaker). Excellent *E* values (>200) were obtained when CALB and acetic anhydride were used in toluene at 60 °C (Scheme 4). The amino alcohols and esters were separated by column chromatography (silica, elution with *n-*hexane:EtOAc 3:1 or 2:1) with high *ee* (≥96%) and good yields (≥43%). Enantiomers (*S*)-**13**–(*S*)-**15** were transformed via methanolysis (in K_2_CO_3_/MeOH at 60 °C) to the corresponding amino alcohols (*S*)-**10**–(*S*)-**12** without a loss in *ee* values (98%) (reactions not shown in the Scheme). MW-assisted Boc deprotection of these products (in water at 100 °C) resulted in the formation of the desired TH*β*C amino alcohols ((*R*)-**16**–(*R*)-**18** and (*S*)-**16**–(*S*)-**18**) without a drop in the *ee* values (≥96%).

In the frame of substrate engineering, the steric effect of different *N*-protecting groups (*N*-Boc, *N*-acetyl, *N*-Cbz, *N*-Fmoc) on the enantioselectivity and reaction rate of CALB-catalyzed *O*-acylation (acetic anhydride, toluene, 60 °C) of 1-hydroxymethyl-TH*β*Cs (±)-**10**, (±)-**19**, (±)-**20**, and (±)-**21** has been investigated [53]. The reactions were carried out in batch mode, in an incubator shaker. Excellent enantioselectivities (*E* > 200) were observed for the acylation of (±)-**10**, (±)-**20**, and (±)-**21** (Scheme 4, Table 1). The resolution of (±)-**19** initially showed excellent *E* (>200), but as the reaction progressed, *E* started to decrease, because of *N*/*O* and *O*/*N* acyl migrations.

Preparative-scale resolutions of (±)-**20** and (±)-**21** resulted in esters (*S*)-**23** and (*S*)-**24** and unreacted amino alcohols (*R*)-**20** and (*R*)-**21** with excellent enantiomeric excess values (≥88%). The products were separated by column chromatography [silica, elution with *n-*hexane:EtOAc (2:1)] (yields *≥* 44%).

- A new total synthesis of crispine A enantiomers via *Burkholderia cepacia* lipase-catalyzed acylation of the primary hydroxy group of *N*-Boc-protected 1-(3-hydroxypropyl)-6,7-bis(methyloxy)-1,2,3,4-THIQ [(±)-**1**] and enantioselective hydrolysis of the corresponding *O*-decanoate [(±)-**2**], with a remote, four-atom-distant stereogenic center was reported [47].
- Both enantiomers of calycotomine [49] and homocalycotomine [50] (one of them is the intermediate of emetine) through *Candida antarctica* lipase B-catalyzed asymmetric *O*-acylation of *N*-Boc-protected (6,7-dimethoxy-1,2,3,4-THIQ-1-yl)methanol ((±)-**4**)), with a remote, two-atom-distant stereogenic center and (6,7-dimethoxy-1,2,3,4-THIQ-1-yl)ethanol ((±)-**7**)), with a remote, three-atom-distant stereogenic center were described.
- A systematic study on the *Candida antarctica* lipase B-catalyzed *O*-acylation of THIQ amino alcohol homologues ((±)-**1**, (±)-**4**, and (±)-**7**) containing a remote stereogenic center was reported [50].
- The synthesis of new enantiomers of 1,2,3,4-TH*β*C derivatives through *Candida antarctica* lipase B-catalyzed *O*-acylation of *N*-Boc-protected 1-hydroxymethyl-1,2,3,4-TH*β*C ((±)-**10**), 1-hydroxymethyl-6-methoxy-1,2,3,4-TH*β*C ((±)-**11**), and 1-hydroxymethyl-6-fluoro-1,2,3,4-TH*β*C ((±)-**12**) was described [52].
- In the frame of substrate engineering, the steric effect of different *N*-protecting groups (*N*-Boc, *N*-acetyl, *N*-Cbz, *N*-Fmoc) on the enantioselectivity and reaction rate of *Candida antarctica* lipase B-catalyzed *O*-acylation of 1-hydroxymethyl-TH*β*Cs [(±)-**10**, (±)-**19**, (±)-**20**, and (±)-**21**] was investigated [53].

#### 2.1.1. KR through *N*-Acylation

A number of racemic cyclic secondary amines bearing a stereogenic center such as 1-substituted 1,2,3,4-THIQs and 1,2,3,4-TH*β*Cs were resolved successfully through *N*-acylation/alkoxycarbonylation. In general, good yields accompanied by high enantioselectivities were obtained. The reactions were performed in an organic solvent either in a continuous-flow system or as a batch reaction.

**Scheme 5:**
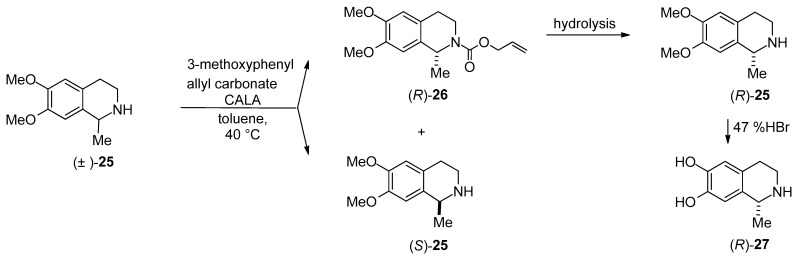
Synthesis of salsolinol enantiomers.

An efficient chemoenzymatic route for the synthesis of (*R*)-salsolinol ((*R*)-**27**) has been described [54]. *Candida antarctica* lipase A (CALA) catalyzed the *N*-acylation (3-methoxyphenyl allyl carbonate, toluene, 40 °C) of 1-methyl-6,7-dimethoxy-1,2,3,4-THIQ (±)-**25** in an enantioselective manner (*E* > 200) resulting in carbamate (*R*)-**26** and unreacted amine (*S*)-**25** with an excellent enantiomeric excess value (98%) and good yield (50%) (Scheme 5). Base-catalyzed hydrolysis (triethanolamine in 50% aqueous NaOH solution at 120 °C) of (*R*)-**26**, followed by treatment with 47% HBr, furnished the desired (*R*)-salsolinol with excellent enantiomeric excess value (98%) and good yield (85%).

**Scheme 6:**
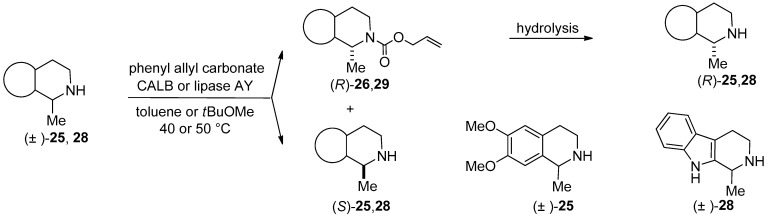
Synthesis of salsolidine enantiomers.

Another enzymatic method for the resolution of salsolidine (±)-**25** and its analogue 1-methyl-1,2,3,4-TH*β*C (±)-**28** through *Candida rugosa* lipase (AY) and CALB-catalyzed enantioselective *N*-alkoxycarbonylation with phenyl allyl carbonate has been reported [55]. Excellent enantioselectivities (*E* > 200) were observed, when the acylation of (±)-**25** was performed in the presence of lipase AY in toluene at 40 °C or with CALB in *t*BuOMe at 50 °C (Scheme 6) in both batch mode and a continuous-flow system. The CALB-catalyzed acylation of (±)-**28**, carried out in the presence of Et_3_N in *t*BuOMe at 50 °C, was also characterized with an excellent enantioselectivity (*E* > 200). The resulting unreacted amine and carbamate enantiomers (*ee* > 97%) were separated by column chromatography (silica, elution with CH_2_Cl_2_:MeOH (1:1) (yields *≥* 44%). The product (*R*)-carbamates [(*R*)-**26**,**29**] (*ee* > 97%) were hydrolyzed into the corresponding free amine (*R*)-enantiomers (*R*)-**25** and (*R*)-**28** (*ee* = 99%) with the use of Pd_2_(dba)_3_.CHCl_3_ as the catalyst.

**Scheme 7:**
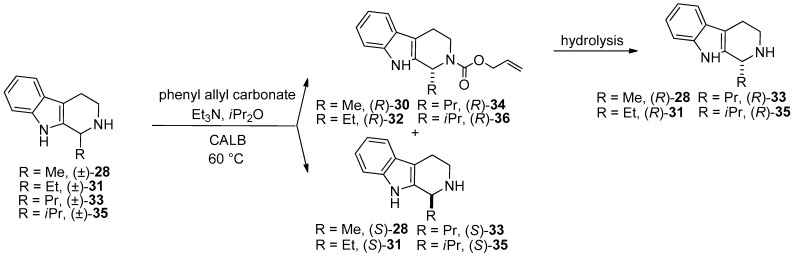
Synthesis of 1-substituted (Me, Et, Pr, *i*Pr) TH*β*C enantiomers.

In the frame of substrate specificity, CALB-catalyzed asymmetric *N*-alkoxycarbonylation of 1-substituted TH*β*Cs (Me, Et, Pr, *i*Pr) has been studied [56]. High enantioselectivities (>200) were observed when alkoxycarbonylations of racemic compounds (±)-**28**,**31**,**33**,**35** were performed in *i*Pr_2_O in the presence of phenyl allyl carbonate and Et_3_N at 60 °C (Scheme 7) resulting in carbamates (*R*)-**30**,**32**,**34**,**36** and unreacted amines (*S*)-**28**,**31**,**33**,**35**. The reaction time had to be increased considerably with increasing substituent size on C1; however, the isopropyl-substituted compound proved to be too bulky to have optimum activity of CALB (Table 2).

Preparative-scale resolutions of (±)-**28**, (±)-**31**, and (±)-**33** resulted in carbamides (*R*)-**30**, (*R*)-**32**, and (*R*)-**34** and unreacted amines (*S*)-**28**, (*S*)-**31**, and (*S*)-**33** with excellent enantiomeric excess values (≥97%). The enantiomeric amines and carbamates were separated by column chromatography (silica, elution with CH_2_ Cl_2_:MeOH (25:1) (yields ≥ 40%). The product (*R*)-carbamates ((*R*)-**32**,**34**) (*ee* = 99%) were hydrolyzed into the corresponding (*R*)-enantiomers of the free amines (*R*)-**31** and (*R*)-**33** (*ee* = 99%, yields > 87%), with the use of Pd_2_(dba)_3_.CHCl_3_ as the catalyst.

#### 2.1.2. DKR through *N*-Acylation

**Scheme 8:**
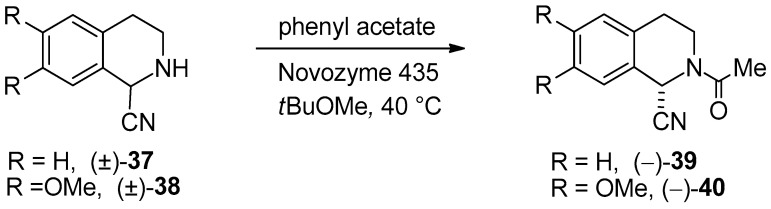
Synthesis of enantiomeric 1-cyano-1,2,3,4-THIQ derivatives.

DKR of 1-cyano-1,2,3,4-THIQ derivatives (±)-**37** and (±)-**38** through lipase (Novozyme 435, PS-C I, PFL and PS)-catalyzed racemization and asymmetric amidation in the presence of phenyl acetate as acyl donor, in *t*BuOMe at 40 °C has been described [57]. When using Novozyme 435 (lipase from *Candida antarctica*) as catalyst, α-amidonitrile products (−)-**39** and (−)-**40** (Scheme 8) were obtained with excellent enantiomeric excess values (*ee* ≥ 95%) and good chemical yields (≥92%). These results showed that dual-function lipase promiscuity resulted in simultaneous racemization and asymmetric amidation of α-aminonitriles (±)-**37** and (±)-**38**, thus providing a useful synthetic method to optically active α-aminonitrile amides (−)-**39** and (−)-**40**.

- Preparation of (*R*)-6,7-dihydroxy-1-methyl-1,2,3,4-THIQ (salsolinol) through *Candida antarctica* lipase A-catalyzed *N*-acylation of 1-methyl-6,7-dimethoxy-1,2,3,4-THIQ [(±)-**25**] was reported [54].
- The synthesis of both enantiomers of salsolidine (the (*R*)-enantiomer has a monoamine oxidase A (MAO A) inhibiting effect) and its analogue 1-methyl-1,2,3,4-TH*β*C carried out through *Candida rugosa* lipase- and *Candida antarctica* lipase B-catalyzed *N*-alkoxycarbonylation of racemic 1-methyl-6,7-dimethoxy-1,2,3,4-THIQ ((±)-**25**) and 1-methyl-1,2,3,4-TH*β*C ((±)-**28**) was described [55].
- In the frame of substrate specificity, the influence of different alkyl chain substituents (Me, Et, Pr, *i*Pr) on the enantioselectivity and reaction rate of *Candida antarctica* lipase B-catalyzed *N*-alkoxycarbonylation of 1-substituted TH*β*Cs (Me ((±)-**28**), Et ((±)-**31**), Pr ((±)-**33**), *i*Pr ((±)-**35**)) was studied [56].
- The synthesis of 1-cyano-1,2,3,4-THIQ enantiomers through *Candida* lipase-catalyzed DKR (racemization and asymmetric amidation) of racemic 1-cyano-1,2,3,4-THIQ (((±)-**39**)) and 1-cyano-6,7-dimethoxy-1,2,3,4-THIQ (((±)-**40**)) was described [57].

### 2.2. DKR through COOEt Hydrolysis

Successful lipase-catalyzed DKR methods were devised for the preparation of enantiomeric 1,2,3,4-THIQ- and 1,2,3,4-TH*β*C-1-CAs with good yield and high enantiomeric excess value through hydrolysis of the hydrochloride salt of 1,2,3,4-THIQ- and 1,2,3,4-TH*β*C-1-CE. The reactions were performed in a buffer solution as a batch reaction. 

**Scheme 9:**
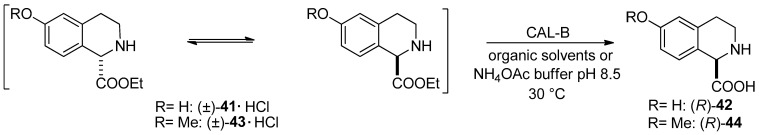
Synthesis of 6-hydroxy- and 6-methoxy-1,2,3,4-THIQ-1-CA enantiomers.

Enantiomeric 6-hydroxy-1,2,3,4-THIQ-1-CA and its 6-methoxy analogue have been synthesized through CALB-catalyzed DKR of the corresponding amino carboxylic ester (CE) hydrochlorides (±)-**41**·HCl and (±)-**43**·HCl (Scheme 9) [58]. Resolution of (±)-**41**·HCl performed in aqueous NH_4_OAc buffer (pH = 8.5) at 30 °C and resolution of (±)-**43**·HCl in organic solvents (*i*Pr_2_O:MeCN (3:2), Pr_2_NH) furnished the desired (1*R*)-6-hydroxy- and (1*R*)-6-methoxy-subsituted 1,2,3,4-THIQ-1-CAs (*R*)-**42**·and (*R*)-**44** with excellent enantiomeric excess values (*ee* > 99%). After filtering the enzyme and evaporating the solvent, (*R*)-**42**·and (*R*)-**44** were isolated with good chemical yields (>87%). (*R*)-**44** can be transformed into (*R*)-2-*tert*-butyl-1-methyl-6-hydroxy-3,4-dihydroisoquinoline-1,2(1*H*)-dicarboxylate, which is the key intermediate of a Liver X Receptor agonist [59]. The *N*-Boc-protected form of racemic 1,2,3,4-TH*β*C-1-CA ((±)-**44**) is a building block in the synthesis of diketopiperazine-fused TH*β*Cs through Ugi four-component reaction [60].

**Scheme 10:**
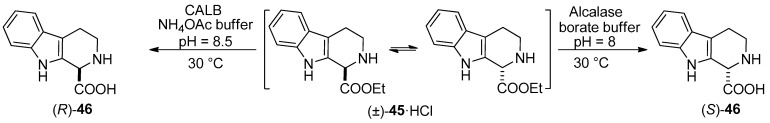
Synthesis of 1,2,3,4-TH*β*C-1-CA enantiomers.

Directed DKR strategies were developed for the preparation of both enantiomers of 1,2,3,4-TH*β*C-1-CA ((*R*)-**46** and (*S*)-**46**) through the hydrolysis of the hydrochloride salt of ethyl 1,2,3,4-TH*β*C-1-carboxylate (±)-**45**·HCl [61]. The CALB-catalyzed reactions, performed in NH_4_OAc aqueous buffer (pH = 8.5) at 30 °C (Scheme 10), gave amino CA (*R*)-**46** with an excellent *ee* of 98%. The solvent was evaporated, the residue dissolved in H_2_O, and the pH 4 set with 5% hydrochloric acid. The solvent was evaporated and (*R*)-**46**·HCl with an *ee* of 98% and a yield of 90% was isolated. The Alcalase (cross-linked immobilized protease aggregation from *Subtilisin Carlsberg*)-catalyzed DKR of (±)-**45**·HCl in borate buffer (pH = 8.0) at 30 °C afforded (*S*)-**46** with an *ee* of 60%. (*S*)-**46**·HCl was obtained with an *ee* of 60% and a yield of 66%.

The authors noted that in (±)-**45**, the ring with the active carboxylate at position 1 is fused to a heteroaromatic pyrrole ring as against a benzene ring, as was the case with tetrahydroisoquinolines (±)-**41**·HCl and (±)-**43**·HCl investigated earlier, which considerably influences the racemization.

- Preparation of (*R*)-6-hydroxy-1,2,3,4-THIQ-1-CA through *Candida antarctica* lipase B-catalyzed DKR of the hydrochloride salt of ethyl 6-hydroxy- and 6-methoxy-1,2,3,4-THIQ-1-carboxylate ((±)-**41**·HCl and (±)-**43**·HCl) was reported. Note that the starting material can be transformed into (*R*)-2-*tert*-butyl-1-methyl-6-hydroxy-3,4-dihydroisoquinoline-1,2(1*H*)-dicarboxylate, which is the key intermediate of a Liver X Receptor agonist [58].
- Both enantiomers of 1,2,3,4-TH*β*C were prepared through *Candida antarctica* lipase B-catalyzed stereocomplementary new DKR processes. The enzymatic hydrolysis of the hydrochloride salts as starting amino CE racemates furnished the desired enantiomeric AAs quantitatively [61].

Presented in Table 3 are the lipase-catalyzed reactions performed in the usable *t*BuOMe and green H_2_O as solvents and those carried out in a continuous-flow system, with clear advantages that include atom economy, reproducibility, safer solvents, short reaction time, rapid heating and compression vs. shaker reactions. 

## 3. Conclusions and Outlook

This compilation presents the most important lipase-catalyzed strategies reported over the last decade for the preparation of pharmaceutically and chemically important THIQ and TH*β*C enantiomers, with simple molecular structure through *O*-acylation of the primary hydroxy group (with remote stereocenter), *N*-acylation of the secondary amino group, and CO*OEt* hydrolysis of the corresponding racemic amino alcohols and amino CEs. 

The strategies are classified as KR and DKR and presented in chronological order. The reactions resulted in the formation of the desired products with excellent *ee* values and good chemical yields isolated either by column chromatography or by organic solvent–H_2_O extraction. The pharmacological importance of the products together with their synthesis is given in the *main text*.

The lipase-catalyzed reactions performed in the usable *t*BuOMe and green H_2_O as solvents and those carried out in a continuous-flow system, with clear advantages including atom economy, reproducibility, safer solvents, short reaction time, rapid heating and compression vs. shaker reactions, are highlighted in the *main text* and summarized in Table 3.

Because of the growing demand for biologically active enantiomeric compounds in pharmaceutical chemistry (e.g., derivatives containing the THIQ or TH*β*C moiety), it is predicted that a large number of new green chemoenzymatic strategies (e.g., flow biocatalysis) will be devised for the preparation of such enantiomers. 

## Figures and Tables

**Table 1:** *O*-acylation of (±)-**10**, (±)-**20**, and (±)-**21**.

Substrate	Reaction Time (min)	*ee*_amino alcohol_ (%) ^b^	*ee*_ester_ (%) ^b^	Conv. (%)	*E*
(±)-**10** *	15	86	99	47	>200
(±)-**20** *	60	69	99	41	>200
(±)-**21** *	60	79	98	45	>200

* 0.0125 mmol substrate; 30 mg mL^−1^ CALB; 2 equiv. acetic anhydride; 1 mL toluene; 60 °C. ^b^ According to HPLC equipped with a Daicel Chiralpak IA or OD-H column.

**Table 2:** *N*-alkoxycarbonylation of (±)-**28**, (±)-**31**, (±)-**33**, and (±)-**35**.

Substrate	Reaction Time (day)	*ee*_amine_ (%) ^b^	*ee*_carbamide_ (%) ^b^	Conv. (%)	*E*
(±)-**28** *	1	99	97	50	>200
(±)-**31** *	3	99	99	50	>200
(±)-**33** *	5	99	99	50	>200
(±)-**35** *	7	27	99	21	>200

* 0.0125 mmol substrate; 30 mg mL^−1^ CALB; 4 equiv. phenyl allyl carbonate; 1 mL *i*Pr_2_O; 10 µL Et_3_N; 60 °C, daily ultrasound shaking. ^b^ According to HPLC equipped with a Chiralpak IA column.

**Table 3:** Green aspects of enzymatic strategies.

Type of the Enzymatic Reaction	Enzymatic Strategies with Green Aspects *	Schemes	Refs
KR through *O*-acylation	Total synthesis of the antitumor-active alkaloid crispine A enantiomers, KRs in *t*BuOMe	Scheme 2	[47]
	Synthesis of calycotomine enantiomers, preliminary experiments of KR in a continuous-flow system	Scheme 3	[49]
	Synthesis of homocalycotomine enantiomers. A systematic study on the *O*-acylation of THIQ amino alcohol homologues containing a remote stereogenic center, in a continuous-flow system	Scheme 3	[50]
	KR of new 1-hydroxymethyl TH*β*C derivatives in batch and continuous-flow systems	Scheme 4	[52]
KR through *N*-acylation	KR of salsolidine and its analogue 1-methyl-1,2,3,4-TH*β*C in *t*BuOMe in a continuous-flow system	Scheme 6	[55]
DKR through *N*-acylation	DKR of 1-cyano-1,2,3,4-THIQ derivatives in *t*BuOMe	Scheme 8	[57]
DKR through CO*OEt* hydrolysis	DKR of 6-hydroxy- and 6-methoxy-1,2,34-THIQ-1-CE hydrochlorides in aqueous NH_4_OAc buffer (pH 8.5)	Scheme 9	[58]
	Directed DKR of 1,2,3,4-TH*β*C-1-CE hydrochloride (in aqueous NH_4_OAc buffer (pH 8.5), borate buffer (pH 8.0))	Scheme 10	[61]

* Reactions in *t*BuOMe and aqueous NH_4_OAc buffer; reactions in continuous-flow system.

## Data Availability

Not applicable.
